# Natural History of Intracranial Atherosclerotic Disease

**DOI:** 10.3389/fneur.2014.00125

**Published:** 2014-07-10

**Authors:** Yuehua Pu, Xin Dou, Liping Liu

**Affiliations:** ^1^Department of Neurology, Beijing Tiantan Hospital of Capital Medical University, Beijing, China

**Keywords:** natural history, intracranial atherosclerosis, stenosis, prognosis, outcome

## Abstract

Intracranial atherosclerotic disease was very common among stroke patients of Asians, Blacks, and Hispanics ancestry. Furthermore, stroke patients with intracranial atherosclerosis (ICAS) have higher recurrence rate of cerebral ischemia and death than those without ICAS. However, the natural history of intracranial atherosclerotic disease is still in controversy. Most of the studies were retrospective and randomized controlled trial of drugs. This review summarized the prognosis of symptomatic and asymptomatic intracranial atherosclerotic disease in order to guide clinical decision-making and further clinical research.

## Introduction

The natural history of disease refers to a description of the uninterrupted progression of a disease in an individual from the moment of exposure to causal agents until recovery or death. Knowledge of the natural history of disease ranks alongside causal understanding in importance for disease prevention and control. It includes four stages: biologic onset, subclinical stage, clinical stage, and outcome. In this review, natural history refers to the clinical outcome under current treatment (including death, recurrent cerebrovascular, or other vascular events, etc.) and regression of intracranial atherosclerotic lesions.

Many studies had confirmed that extracranial carotid atherosclerotic stenosis was the most common vascular lesion found in stroke patients in Caucasians, otherwise intracranial atherosclerotic disease was found commonly among stroke patients of Asians, Blacks, and Hispanics ancestry (1–3). Stroke patients with intracranial atherosclerosis (ICAS) have higher recurrence rate of cerebral infarct event and death. Owing to heterogeneity in vascular anatomy and physiology, atherosclerosis in different vessels may represent diseases with fundamentally distinct courses. Therefore, it is important to distinguish vascular territories when studying the natural history of this condition (4).

## Progression of Cerebral Atherosclerotic Lesions

Atherosclerosis is a progressive disease that starts early in life and is manifested clinically as coronary heart disease (CHD), cerebrovascular disorders, or peripheral arterial disease (5). This disease can be hidden in the human body for many years and ultimately lead to vascular remodeling. Glagov and co-workers (6) put forward the concept of vascular remodeling early in the 80s of the last century. They held that it leads to expansion of the vascular wall at first and does not influence diameter of lumen and supply of blood in the early atherosclerosis, and then to late, it leads to stenosis of blood vessel diameter, thus it causes changes in hemodynamics and even occlusion. Early in 1994, Schwarze et al. (7) had found that intracranial arterial stenoses are dynamic lesions, and that they can evolve and cause further reductions of the arterial diameters after relatively short periods of time. They observed a group of patients with a mean follow-up of 21 months. Ten (35%) arteries with lesions had TCD evidence of progression.

Wong and his colleagues (8) observed 143 patients with symptomatic middle cerebral artery (MCA) stenosis or occlusion. They repeated TCD examinations 6 months after the initial examinations and recorded any stroke or coronary events during this period. The changes of MCA flow velocities were categorized as normalized artery, stable artery, and progressed artery, which were determined according to the changes of MCA velocities at 6 months. By analyzing both the initial and repeated TCD findings, there were 42 patients (29%) in the normalized group, 88 patients (62%) in the stable group, and 13 patients (9%) in the progressed group. For the clinical events during the 6-month period, 18(12.6%) of the patients had further documented vascular events, including 10 recurrent strokes (9 ischemic strokes and 1 hemorrhagic stroke), 5 TIAs, and 3 acute coronary syndromes. Progression of MCA occlusive diseases is associated with an increased risk of vascular events. Jeon et al. (9) studied 103 patients with MCA stenosis (include symptomatic and asymptomatic). To understand its progress situation, patients need to take the TCD reexamination at 6 months after the initial examination. After 6 months, 13 (12.6%) patients showed worsening, whereas 90 were stationary or showed regression upon TCD examination. In this study, the definition of the progression of MCA stenosis as determined TCD examination was not the same as Wong’s.

So, intracranial artery stenosis is a process of dynamic changes. The speed of progress is different from each other. Through the prospective study, it is found that lesions may progress, improve, or no change over a period of time. The proportion of progression is about 9–12% for 6 months. The possibility of the progression depends on the time, and it may be greater as time goes on. The progression of stenosis may lead to increased risk of vascular events. However, there is a lack of large sample studies in this area, and the risk factors leading to advances in intracranial artery stenosis still remain to be determined. Most of the present studies are based on TCD. This examination method needs to a higher requirement for operators and exists to some limitations. In the future, it is urgent to develop the studies based on MRA, CTA, or DSA.

## The Clinical Outcome of Intracranial Artery Stenosis

Major trials investigating prognosis of asymptomatic and symptomatic intracranial large artery stenosis or occlusion disease was shown in Table 1.

**Table 1:** **Major trials investigating prognosis of asymptomatic and symptomatic intracranial large artery stenosis or occlusion disease**.

Reference	Design	No. of patients	Mean follow-up (Month)	Degree of stenosis (%), diagnosis method	Outcome Assessment	Prognosis
Kern et al. (10) (For asymptomatic MCA)	Prospective, observational	102	30.7	TCD and TCCD	Cerebrovascular events, including TIA and stroke	Overall stroke risk was 12.5% per year (ipsilateral: 9.1%) for patients with symptomatic MCA disease the annual; that of asymptomatic MCA disease was only 2.8% (ipsilateral: 1.4%).
Wong and Li (11)	Prospective, observational	705	42	>50%, by TCD	Further vascular events (including TIA, stroke, or acute coronary syndrome) or death	Annual risk of death: 11.2%. Annual risk of cerebrovascular event: 17.1% (For patients only have intracranial stenosis or occlusion)
Chimowitz et al. (12)	Randomized, double-blinded, multicenter trial	569	21.6	50~99%, by DSA	The primary end point: ischemic stroke, brain hemorrhage, or death from vascular causes other than stroke	The primary end point occurred in 22.1% of the patients in the aspirin group and 21.8% of those in the warfarin group.
Mazighi et al. (13)	Prospective, multicenter, non-randomized	102	23.4	50~99%, by DSA or ultrasonography and confirmed by MRA, angiography, or CT	Cerebrovascular event: ischemic stroke and TIA, or vascular death.	The overall vascular death rate was 8.8%. The rate of patients had a cerebrovascular event was 38.2%.
Chimowitz et al. (14)	Investigator-initiated, randomized, clinical trial	451	11.9	50~99%, by angiography	The primary end point: stroke or death within 30 days or stroke in the territory of the qualifying artery beyond 30 days.	1-year rates of the primary end point was 20.0% in the PTAS group and 12.2% in the medical-management group.
Miao et al. (15)	prospective, randomized, controlled, single-center	70	12	Symptomatic MCA stenosis ≥70%, by DSA	The end point events: any kind of ipsilateral stroke or transient ischemic attack, or death from any origin	30-day rate of end point events was 8.3% for PTAS group and 5.9% for medical group. One-year rate of end point events was 19.4 and 17.6%.

*TIA, transient ischemic attack; TCD, transcranial Doppler; TCCD, transcranial color-coded duplex ultrasonography; PSV, peak systolic flow velocity; DSA, digital subtraction angiography; MCA, middle cerebral artery; PTAS, percutaneous transluminal angioplasty and stenting; MRA, magnetic resonance angiography; CT, computer tomography; WASID: warfarin–aspirin symptomatic intracranial disease; GESICA: Groupe d’Etude des stenoses intra-craniennes atheromateuses symptomatiques; SAMMPRIS, stenting and aggressive medical management for preventing recurrent stroke in intracranial stenosis*.

### Asymptomatic intracranial artery stenosis

Kremer and co-workers (16) follow-up 50 white patients with asymptomatic atherosclerotic middle cerebral artery stenosis (MCAS) as determined TCD examination for [mean (SD)] 815(351) days. The results showed that no patient suffered an ischemic event in the MCAS territory; one had a transient ischemic attack (TIA) in the contralateral hemisphere; three patients died (one from a subdural hematoma in the contralateral hemisphere, and two from non-stroke-related causes) during the follow-up period. So they came to the conclusions that asymptomatic MCAS of atherosclerotic origin appears to have a benign long-term prognosis with a low risk of ipsilateral stroke in medically treated white patients. Kern et al. (10) took the comparison of the symptomatic and asymptomatic MCA stenosis in patients with recurrent stroke risk and came to a similar conclusion. The authors observed 102 consecutive patients with significant MCA stenosis or occlusion as demonstrated by transcranial Doppler and transcranial color-coded duplex ultrasonography. Patients with symptomatic MCA disease had an overall stroke risk of 12.5% per year (ipsilateral: 9.1%), whereas the annual incidence in primarily asymptomatic MCA disease was only 2.8% (ipsilateral: 1.4%; *p* < 0.01). It was significantly lower than that of symptomatic MCA stenosis. Symptomatic MCA disease was an independent predictor for overall [hazard ratio (HR)] 7.91, 95% CI 2.03–30.79; *p* < 0.01) and ipsilateral (HR 9.66, 95% CI 1.5–62.25; *p* = 0.02) cerebrovascular events. Borozan et al. (17) made a retrospective study for patients with intracranial ICA stenosis with mean follow-up of 25.5 months, 93 patients were included, and 24% were symptomatic. Patients were considered for inclusion in this study if a carotid siphon stenosis of 20% or greater was documented arteriographically, and they were identified from a review of 885 consecutive cerebral arteriograms. In this study, overall annual rate of stroke per year was 5.1%, annual incidence of ipsilateral stroke was 5.0%, and overall mortality was 10.6%. Obviously, subgroup analyses revealed that for patients with symptomatic stenosis, annual incidence of ipsilateral stroke per year was 6.4%, whereas that of patients with asymptomatic stenosis was 3.5%.

### Symptomatic intracranial artery stenosis

Many studies have confirmed that there is a higher risk of death and vascular events in patients with ICAS. Wong et al. (11) included 705 patients with acute ischemic stroke and followed up for 42 months. The annual recurrent stroke rates during the first year were 10.9% for patients without vascular lesion, 17.1% for ICAS only, and 24.3% for both intracranial and extracranial atherosclerosis; for the second year, the rates were 7.5, 8.6, and 7.7%, respectively. More occurrence of death (log rank, 5.19; *p* = 0.02) or cerebrovascular event (log rank, 9.68; *p* = 0.002) was found among patients with than those without vascular lesions. Patients with both intracranial and extracranial arterial lesions were at highest risk of death (log rank, 9.64; *p* = 0.008) and cerebrovascular event (log rank, 11.56; *p* = 0.003). They came to the conclusions that patients with ICAS, especially co-existing extracranial carotid disease, are at higher risk of suffering death or further vascular event.

The GESICA study (13) was another prospective, multicenter, non-randomized study from France. The objective was to evaluate the natural history of ICAS and, in those patients refractory to medical treatment, the outcomes associated with intracranial angioplasty. Patients aged 18–80 were enrolled with symptoms attributed to a single ICAS of greater than or equal to 50%. Optimal medical therapy of vascular risk factors and preventive antithrombotic therapy were at the discretion of the local investigator. Intracranial stenosis (50–99%) had to be demonstrated by either DSA or ultrasonography and confirmed by one of the following methods: MRA, angiography, or CT. During a mean follow-up of 23.4 months, 38.2% of the patients had a cerebrovascular event: ischemic stroke in 13.7%, and TIA in 24.5%. Cardiovascular events occurred in 18.6% of patients. The overall vascular death rate was 8.8%. At the same time, the study also held that clinically significant hemodynamic stenoses were associated with stroke recurrence and may help identify a high-risk subset of patients.

Specific medicine was still not found for ICAS patients. Antithrombotic therapy for intracranial arterial stenosis was evaluated in the Warfarin versus Aspirin for Symptomatic Intracranial Disease (WASID) trial (18). They reported the risk of stroke in the territory of the stenotic artery was highest with severe stenosis ≥70% (HR 2.03; 95% CI 1.29–3.22; *p* < 0.0025). In the NIH registry on use of the Wingspan stent for symptomatic 70–99% intracranial arterial stenosis (19), comparison of the event rates in high-risk patients in WASID do not rule out either that stenting could be associated with a substantial relative risk reduction (e.g., 50%) or has no advantage compared with medical therapy. The frequency of any stroke, intracerebral hemorrhage, or death within 30 days or ipsilateral stroke beyond 30 days was 14.0% at 6 months (95% CI = 8.7–22.1%). The frequency of ≥50% restenosis on follow-up angiography was 13/52 (25%). The SAMMPRIS (14) study compared percutaneous transluminal angioplasty and stenting (PTAS) with medical management to prevent recurrent stroke. One-year rates of the primary end point (stroke or death within 30 days after enrollment or after a revascularization procedure) were 20.0% in the PTAS group and 12.2% in the medical-management group.

In the Chinese IntraCranial AtheroSclerosis (CICAS) study (20), we evaluated 2864 consecutive patients who experienced an acute cerebral ischemia within 7 days of symptom onset. The prevalence of ICAS was 46.6% (1,335 patients, including 261 patients with co-existing extracranial carotid stenosis). Patients with ICAS had more severe stroke at admission and stayed longer in hospitals than those without intracranial stenosis (median NIHSS 3 vs. 5; median length of stay 14 vs. 16 days, respectively, both *p* < 0.0001). After 12 months, recurrent stroke occurred in 3.34% of patients with no stenosis, 3.82% for 50–69% stenosis, 5.16% for 70–99% stenosis, and 7.40% for total occlusion.

### Clinical outcomes of ICAS by different vessels

The clinical outcomes of ICAS may depend on the situation of vessels (21). A multicenter prospective study from German showed the prognosis of basilar artery occlusion was worse than carotid artery and MCA occlusion (22). The location of lesions may affect prognosis and treatment. It is necessary to take a review to natural history based on distribution of ICAS.

#### Intracranial internal carotid artery

In 1982, Craig et al. (23) published their first retrospective study and explored the natural history of intracranial internal carotid artery (ICA) atherosclerosis. The diagnosis was confirmed by DSA to demonstrate that stenosis rate >30%. The results suggested that overall annual incidence of stroke was 11.6%; annual incidence of ipsilateral stroke events was 7.6%; symptomatic patients is higher than that in patients with asymptomatic stenosis in annual recurrence rate of cerebral ischemia events (45 vs. 36%); but there was no differences between the two groups in overall mortality (42 vs. 45%). In the same year, Marzewki et al. (24) published a similar retrospective case series study on intracranial ICA stenosis. The diagnosis was confirmed by angiography that stenosis rate >50%. Overall annual recurrence rate of stroke per year was 3.9%; incidence of ipsilateral stroke was 3.1%. Bogousslavsky (25) observed 22 patients with stenosis rate greater than 30% documented by angiography. During a mean follow-up of 40.4 months, seven patients died (32%), and among them, six patients died of heart disease and one died of stroke. In this study, annual incidence of ipsilateral stroke per year was 8.1% and the annual mortality was 9.5%.

#### Middle cerebral artery

Although most of previous studies were miniature retrospective studies, recently, more and more countries and regions successively carried out a number of large-size prospective studies. These studies reported that average annual mortality and annual incidence of ipsilateral stroke were 2.7–7.1%. Furthermore, these studies also suggested that progression of plaque and appearance of Microembolic signals (MES) often foreshowed stroke recurrence. So these methods were helpful to screen high-risk patients. For studies on MCA, they also began from a series of small-sample retrospective case studies, which was similar to the related studies on intracranial ICA and vertebrobasilar artery (VB). The first paper published in 1979. Hinton et al. (26) analyzed 16 consecutive patients with symptomatic MCA stenosis. Follow-up was from 1 month to 6 years, and finally 2 of the 16 (12.5%) developed severe stroke events, which were located in the territory of stenotic MCA, no deaths. Corston et al. (27) reviewed 21 patients with symptomatic intracranial artery stenosis confirmed by angiography, and among them, 90% had M1 segment of MCA lesions. During the mean follow-up period of 6.5 years, 5 patients (24%) suffered stroke events (4 of them were fatal stroke), 10 patients (48%) died due to various reasons. Annual incidence of stroke was 3.7%, overall annual mortality was 7.1%, and annual incidence of fatal stroke was 2.9%.

The first prospective study about MCA stenosis was from a subgroup analysis of intracranial and extracranial bypass (28). This study was about the efficacy of drug therapy and extracranial–intracranial bypass operation for ICAS. In the group of drug therapy, 138 patients with symptomatic MCA stenosis or occlusion were recruited. During the mean follow-up period of 55.8 months, 23% of patients suffered stroke events in the territory of arbitrary vessels and overall annual incidence of stroke was 5.0%. This study did not report location and mortality of stroke. Arenillas et al. (29) conducted a prospective study in view of symptomatic MCA stenosis. They screened from consecutive TIA or stroke patients and 40 of them entered this study, which confirmed by TCD and cerebral angiography as MCA stenosis. During the mean follow-up period of 26.5 months, eight patients (20%) occurred cerebral ischemic event in the territory of stenotic MCA (six TIAs and two stokes), annual incidence of ipsilateral TIA was 6.8%; annual incidence of ipsilateral stroke was 2.3%. Progression of stenosis was independent predictors of stroke recurrence. The results of this study suggested that periodic review of transcranial Doppler ultrasound was helpful to screen patients with high-risk. Gao et al. (30) provided another method to identify high-risk population for us. This prospective study observed 114 consecutive patients with acute ischemic stroke. These patients were confirmed by TCD as MCA stenosis. Each patient received TCD examination for three consecutive days during acute period, 30 min for each time, which detected the existence of MES. During the mean follow-up period of 13.6 months, 10 patients recurred stroke in the territory of stenotic MCA and 9 patients died. Annual recurrence rate of ipsilateral stroke was 7.8%; overall annual mortality was 7.0%. TCD found that MES was independent predictors of stroke recurrence. This study not only indicated mechanism of embolization in stroke recurrence, but also showed a new method to confirm patients at high-risk – MES detection. Recently, Miao et al. (15) performed a prospective, randomized, controlled, and single-center clinical trial to compare PTAS with medical treatment for symptomatic MCA stenosis (≥70%). The PTAS group received stenting or balloon angioplasty, whereas the medical treatment group received standard medical treatment (aspirin 100 mg plus clopidogrel 75 mg/day), and all the patients were under strict control of the risk factors. The end point events were any kind of ipsilateral stroke or TIA, or death from any origin during 1-year follow-up. The 1-year rate of end point events was 19.4 vs. 17.6% (*p* = 0.85) for PTAS and medical group, respectively.

#### Vertebrobasilar artery

Researchers in WASID carried on a investigation, which prompted that in the case of intracranial artery stenosis, the involved vertebral basilar artery was 35–40% (12). There were also many limitations in studies of outcomes of posterior circulation atherosclerosis. There exist obvious variation in the average annual stroke recurrence rate in territory of stenosis artery, overall annual incidence of stroke, overall annual mortality in these studies, and range of which was 0–8.7%, 3.0–14.3%, and 2.9–42.8%.

The subgroup analysis from warfarin–aspirin symptomatic intracranial disease (WAISD) (31) study group showed that 22% of patients had an ischemic stroke (arbitrary territory of artery), 15% of patients suffered stroke in the territory of the stenotic artery, and 10.3% of patients died (5.9% of them died of stroke). These results suggested that annual mortality was 6.2%, overall incidence of stroke was 13.1% and incidence of stroke in the territory of vertebral basilar artery was 8.7%. According to the location of specific plaque, they assessed the recurrence rate of stroke. For patients with vertebral artery stenosis, annual incidence of stroke in the territory of the stenotic artery was 7.8%, whereas for patients with basilar artery stenosis, incidence of stroke was 10.7%. For posterior cerebral artery and posterior inferior cerebellar artery, the incidence of stroke was 6.0%.

Qureshi et al. (32) published a retrospective multicenter study. A total of 102 patients were included, which accepted the mean follow-up period of 15 months. Fourteen patients experienced recurrent stroke (arbitrary territory of artery). Eight patients experienced stroke in the territory of vertebral basilar artery. Twenty-one patients died during follow-up, and among them, 16 patients died of fatal stroke. These results suggested that overall incidence of stroke was 11%, annual incidence of stroke in the territory of vertebral basilar artery was 6.3%, and overall annual mortality was 6.3%. Kaplan–Meier analysis revealed that stroke-free survival of patients was 76% at 12 months and 48% at 5 years. This suggested that most of patients with symptomatic intracranial VB stenosis, in the 5 years after the initial onset, would suffer recurrent stroke or death. In this analysis, elderly and lack of antiplatelet or anticoagulant therapy are independent predictors of poor prognosis.

A series of reports from New England Medical Center Posterior Circulation registry made us better understanding the stroke in posterior Circulation. The overall 30-day mortality was 3.6%. Embolic mechanism, distal territory location, and basilar artery-occlusive disease carried the poorest prognosis. The best outcome was in patients who had multiple arterial occlusive sites; they had position-sensitive TIAs during months to years (33). For patients with moderate to severe BA occlusive disease, the mortality rate was 2.3%, and 62 patients (almost 75%) had minor or no deficits at follow-up (34). For patients with bilateral ICVA (intracranial vertebral artery) occlusive disease, the short- and long-term (mean length of follow-up was 31.4 months) outcomes were usually favorable, but patients with bilateral ICVA and basilar artery-occlusive lesions often have poor outcomes (35). The patients with distal territory infarcts due to emboli from the ICVA had the worst outcome (36).

## Summary and Expectation

Overall, intracranial arterial stenoses are dynamic lesions, and that they can evolve and cause further reductions of the arterial diameters or further vascular events as time goes on. However, it is still uncertain that how to recognize high-risk population and what methods can prevent further vascular event efficiently. On the other hand, the natural history of symptomatic and asymptomatic disease is different. The annual incidence of ipsilateral stroke per year for patients with asymptomatic stenosis was much lower than that of patients with symptomatic stenosis. Asymptomatic and symptomatic lesions can transform for each other. But, we do not yet know how this conversion is made. Furthermore, location of the diseased vessel may affect prognosis and treatment. There still lack of large sample, prospective, and observational study to identify the natural history of intracranial large artery disease by different vessels.

## Conflict of Interest Statement

The authors declare that the research was conducted in the absence of any commercial or financial relationships that could be construed as a potential conflict of interest.
